# CSF1R Ligands IL-34 and CSF1 Are Differentially Required for Microglia Development and Maintenance in White and Gray Matter Brain Regions

**DOI:** 10.3389/fimmu.2019.02199

**Published:** 2019-09-20

**Authors:** Courtney Easley-Neal, Oded Foreman, Neeraj Sharma, Ali A. Zarrin, Robby M Weimer

**Affiliations:** ^1^Department of Biomedical Imaging, Genentech, Inc., South San Francisco, CA, United States; ^2^Department of Pathology, Genentech, Inc., South San Francisco, CA, United States; ^3^Department of Immunology, Genentech, Inc., South San Francisco, CA, United States

**Keywords:** Csf1r, Csf1, IL-34, microglia, CNS

## Abstract

Microglia are specialized brain macrophages that play numerous roles in tissue homeostasis and response to injury. Colony stimulating factor 1 receptor (CSF1R) is a receptor tyrosine kinase required for the development, maintenance, and proliferation of microglia. Here we show that in adult mice peripheral dosing of function-blocking antibodies to the two known ligands of CSF1R, CSF1, and IL-34, can deplete microglia differentially in white and gray matter regions of the brain, respectively. The regional patterns of depletion correspond to the differential expression of CSF1 and IL-34. In addition, we show that while CSF1 is required to establish microglia in the developing embryo, both CSF1 and IL-34 are required beginning in early postnatal development. These results not only clarify the roles of CSF1 and IL-34 in microglia maintenance, but also suggest that signaling through these two ligands might support distinct sub-populations of microglia, an insight that may impact drug development for neurodegenerative and other diseases.

## Introduction

Microglia, the resident immune cells of the brain, are a type of specialized macrophage that plays numerous roles in tissue homeostasis and response to injury (1–3). They are derived in the yolk sac from macrophage progenitors early in embryonic development, and migrate to and colonize the developing brain (4, 5). In many ways microglia function as classic tissue resident macrophages, acting as phagocytes in clearing cellular debris, releasing cytokines such as tumor necrosis factor-α (TNF-α) and interleukin-6 (IL-6), and releasing nitric oxide (NO) (6, 7). In recent years microglia have come to be appreciated as playing a broad set of roles beyond their immunological functions, including roles in establishing brain architecture and wiring during development, postnatal synapse refinement, adult neurogenesis, and learning-dependent synapse formation and elimination (8–13).

Colony stimulating factor 1 receptor (CSF1R) is a class III receptor tyrosine kinase, and CSF1R signaling is required for the development, survival, recruitment and proliferation of mononuclear phagocytes, including microglia and macrophages (14, 15). CSF1R is expressed in all macrophages and monocytes as well as osteoclasts (16). CSF1R has two known ligands, CSF1 and IL-34, which have low amino acid sequence identity but very similar tertiary structure, and have overlapping but distinct binding sites on CSF1R (17–19). In the brain, CSF1 is primarily expressed by astrocytes, oligodendrocytes, and microglia, while IL-34 is predominantly expressed by neurons (20, 21). Mice lacking CSF1R or one of its ligands have reduced microglia density in the brain and defects in other monocyte populations throughout the body (22–27). The *CSF1R* knockout mouse is missing almost all microglia at 3 weeks of age, while in adult *CSF1* null and *IL-34* null mice varying degrees of microglia depletion are seen in different brain regions, and the degree of microglia depletion changes from early postnatal development to adulthood (5, 22–24).

While much has been learned about the role of CSF1R in microglia development and maintenance in the brain from CSF1R signaling-deficient mice, it is still unclear what the contributions of CSF1 and IL-34 are to this process. One way to tease apart their spatial and temporal roles is to block the function of CSF1 and IL-34 during development or in adult mice and measure the effect on microglia throughout the brain. Function-blocking antibodies are a potential approach to do this as they can be dosed at any time point and they specifically bind to a ligand and prevent its interaction with, and downstream signaling via, its target receptor. Additionally, they provide the benefit of not having to generate conditional knockout animals to assess the role of CSF1 and IL-34 during different phases of development and in the adult (28).

In this study we demonstrated that in adult animals, peripherally dosed function-blocking antibodies to CSF1 and IL-34 deplete microglia in the brain. Anti-CSF1 is most effective in depleting white matter microglia, while anti-IL-34 is most effective in depleting gray matter microglia—a regional pattern coincident with the expression of each ligand. Microglia depletion was dose-dependent, with higher doses of antibody required to deplete microglia than peripheral macrophages, suggesting that the antibodies engage their ligands within the CNS to mediate this effect. Furthermore, dosing of anti-CSF1 and anti-IL-34 during pre or postnatal development revealed that only CSF1 is required for microglia colonization and maintenance in the embryonic brain, IL-34 begins to be required for microglial maintenance in postnatal life, and the adult pattern of gray and white matter specific ligand dependence does not emerge until a later point in development. Taken together, these results suggest distinct requirements for CSF1 and IL-34 in the development and maintenance of microglia in the CNS.

## Results

### IL-34 Is Required for Microglia Maintenance in Gray Matter and CSF1 Is Required for Microglia Maintenance in White Matter in Adult Mice

**Figure 1:**
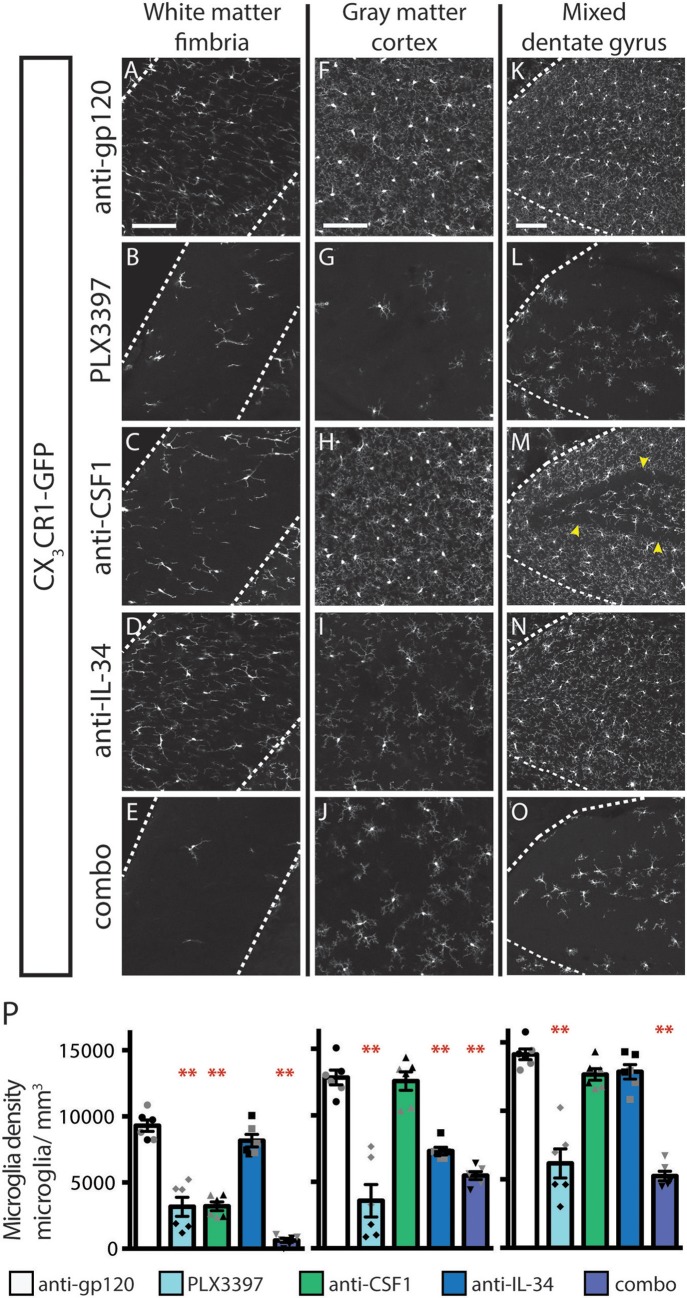
Dosing with function-blocking antibodies to CSF1R ligands IL-34 and CSF1 results in differential microglial depletion in white and gray matter. **(A–O)** Representative images of microglia in brains from adult *CX*_3_*CR1*^*GFPki*/+^ mice treated with anti-gp120 (control IgG), anti-IL-34, anti-CSF1, combo (anti-IL-34 plus anti-CSF1) dosed IP, 2X per week for 3 weeks at 100 mg/kg each or PLX3397 (formulated in chow at 290 mg/kg for 3 weeks). Images of fimbria (**A–E**; white matter; outlined by dashed white lines), somatosensory cortex layers I-III (**F–J**; gray matter), and dentate gyrus (**K–O**; outlined by dashed white lines). **(M)** Granule cell layer indicated by yellow arrowheads. **(P)** Quantification of microglia density in fimbria, cortex, and dentate gyrus. *n* = 6 animals/group, 3 males and 3 females. Males indicated by black symbols, females by gray symbols. Data are represented as mean ± SEM, ^**^ indicates *p* < 0.0001. Data were analyzed with a one-way ANOVA with a *post-hoc* Dunnett's test. Scale bars, 100 μm.

Inhibition of CSF1R signaling in adult animals, *via* dosing with CSF1R small molecule inhibitors (SMI), such as PLX3397, results in the widespread loss of microglia throughout the brain (29, 30). To assess if either CSF1 or IL-34 is required for the maintenance of microglia populations in adult animals, we utilized function-blocking antibodies that could specifically bind CSF1 (anti-CSF1) or IL-34 (anti-IL-34) and prevent binding to CSF1R, without disrupting the interaction of the receptor with the other ligand (31). Two-month-old adult mice heterozygous for the CX_3_CR1-GFP allele (*CX*_3_*CR1*^*GFPki*/+^) (32) were dosed with anti-gp120 (control IgG), anti-IL-34, anti-CSF1, a combination of anti-CSF1 and anti-IL-34 (combo), or PLX3397 for 3 weeks. Similar to published observations, dosing with PLX3397 resulted in widespread loss of GFP+ microglia (Figure 1 and Figure S1). In contrast, dosing with anti-CSF1 alone resulted in significant depletion in white matter tracts such as the hippocampal fimbria and the corpus callosum (Figures 1A–E,P and Figure S1), but no depletion in gray matter regions such as the cortex and the striatum (Figures 1F–J,P and Figure S1). Conversely, anti-IL-34 dosing significantly depleted microglia in gray matter, such as the cortex and the striatum (Figures 1F–J,P and Figure S1), but did not deplete microglia in white matter tracts such as the fimbria or corpus callosum (Figures 1A–E,P and Figure S1). Mixed brain regions composed of gray matter and axon bundles, such as the dentate gyrus (DG) and cerebellum, had differential responses to anti-CSF1, with no depletion in the DG but significant depletion in the cerebellum (Figures 1K–O,P and Figure S1). Both DG and cerebellum showed no significant reduction in microglia density with anti-IL-34 dosed singly, but combo dosing did significantly deplete microglia in both brain regions (Figures 1K–O,P and Figure S1). Interestingly, anti-CSF1 specifically depleted microglia in the granule cell layer of the DG (Figure 1M, arrowheads). No other cell body layers, such as CA1 or CA3, showed depletion with anti-CSF1 or anti-IL-34 dosing. In all brain regions examined, combo dosing resulted in increased microglia depletion compared to singly dosed antibodies (Figures 1E,J,O,P, and Figure S1), suggesting that the non-dominant ligand can compensate to some extent when the dominant ligand is blocked.

A similar pattern of depletion is seen in the spinal cord, with anti-CSF1 significantly depleting microglia in the white matter of the dorsal column and anti-IL-34 significantly depleting microglia in the gray matter of the dorsal horn (Figure S2). Combo dosing did not enhance depletion in the white matter but did lead to increased depletion in the gray matter (Figures S2B, S2C).

Dosing with PLX3397 was used as a positive control for microglia depletion in adult mice. Male PLX3397 dosed mice had a similar or greater degree of microglia depletion compared to combo-dosed mice, depleting 71–90% of microglia in all brain regions examined. Surprisingly, female PLX3397 dosed animals showed significantly less depletion (41–68%; Figure 1P, Figure S3A). Pharmacokinetic (PK) data from these mice shows that males have an equivalent degree of depletion at tissue concentrations of 6.6 μM PLX3397 and above (R^2^ = 0.007), while females showed tissue concentration-dependent depletion, over a similar range of PLX3397 tissue concentrations (R^2^ = 0.318; Figure S3B). This gender-driven difference in depletion is specific to PLX3397, as dosing with anti-IL-34 and/or anti-CSF1 leads to equivalent degrees of microglia depletion in both male and female mice in all brain regions assessed (Figure 1P, males: black symbols, females: gray symbols, and Figure S1). This suggests a sex-specific difference in response to PLX3397 rather than sex-specific effects from targeting CSF1R signaling.

### Dose-Ranging Suggests That Anti-CSF1 and Anti-IL-34 Engage Their Targets in the Brain

**Figure 2:**
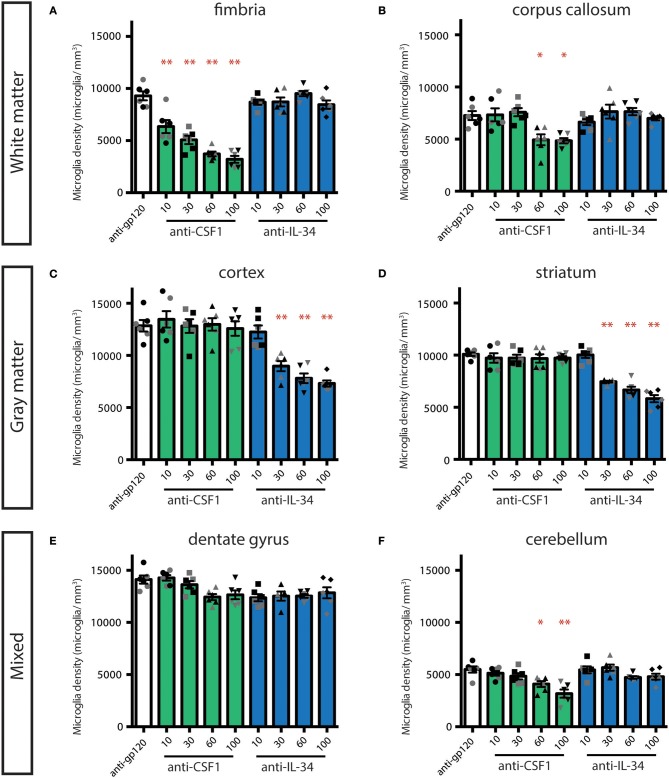
Dose-dependent depletion of brain microglia with peripherally dosed anti-IL-34 and anti-CSF1. **(A–F)** Quantification of microglia density in adult *CX*_3_*CR1*^*GFPki*/+^ mice dosed with anti-gp120 100 mg/kg, anti-IL-34 ranging from 10 to 100 mg/kg, or anti-CSF1 ranging from 10 to 100 mg/kg. Mice were dosed IP, 2X per week for 3 weeks. **(A,B)** Histograms of microglia density in hippocampal fimbria and corpus callosum, white matter regions. **(C,D)** Histograms of microglia density in striatum and cortex, gray matter regions. **(E,F)** Histograms of microglia density in dentate gyrus and cerebellum, mixed brain regions. *n* = 6 animals/group, 3 males and 3 females. Males indicated by black symbols, females by gray symbols. Data are represented as mean ± SEM, ^*^p < 0.05, ^**^p < 0.001. Data were analyzed with a one-way ANOVA with a *post-hoc* Dunnett's test.

In our initial study, we assessed the efficacy of high doses of antibodies with the understanding that only a small portion of peripherally dosed antibody crosses the blood-brain barrier (33). To further evaluate whether the effect of peripherally dosed anti-CSF1 and anti-IL-34 antibodies on microglia density was due to peripheral or central ligand inhibition, we conducted a dose-ranging study in *CX*_3_*CR1*^*GFPki*/+^ animals, starting with doses previously shown to inhibit ligand activity in the periphery (10 mg/kg) (31). This study shares the control-dosed group with the study presented in Figure 1, and the anti-CSF1 and anti-IL-34 100 mg/kg data are re-stated for comparison with the lower dosing groups. The fimbria was the only region to show significant microglia depletion with a low dose of either antibody, with anti-CSF1 significantly depleting microglia at 10 mg/kg (Figure 2A; 10 mg/kg: 22%, 30 mg/kg: 45%, 60 mg/kg: 60% and 100 mg/kg: 65% depletion), while in the corpus callosum only high doses of anti-CSF1 resulted in depletion (Figure 2B; 60 mg/kg: 32% and 100 mg/kg: 33% depletion). In the cortex, anti-IL-34 depleted microglia in a dose-dependent manner, with 10 mg/kg causing no depletion, and increasing doses causing increasing levels of depletion (Figure 2C, 30 mg/kg: 30%, 60 mg/kg: 39%, 100 mg/kg: 43% depletion). This pattern was repeated in the striatum (Figure 2D; 30 mg/kg: 24%, 60 mg/kg: 34%, 100 mg/kg: 42% depletion). In the dentate gyrus there was a small, non-significant reduction of ~10% in microglia density with all doses of anti-IL-34 and with high doses of anti-CSF1 (Figure 2E). And in the cerebellum there was a small, non-significant reduction of ~12% with high doses of anti-IL-34 and a significant reduction of microglia density with high dose anti-CSF1 (Figure 2F; 60 mg/kg: 25%, 100 mg/kg: 42%). In all regions evaluated, there were no observed differences in the degree of microglia depletion between male and female treated with anti-CSF1 or anti-IL-34 antibodies (*n* = 3M, 3F; *p* > 0.5), consistent with results from the previous study. Other than in the fimbria, low dose anti-IL-34 and anti-CSF1 fail to deplete microglia in the brain, while they effectively deplete peripheral macrophages dependent on CS1FR signaling (Figure 2, (31)). This result is consistent with microglia depletion being a central effect of the antibodies crossing the blood-brain barrier and not a peripheral effect on CSF1R signaling.

Additionally, the degree of depletion seen with 60 and 100 mg/kg dosing appears to phenocopy microglia loss seen in the *CSF1* null and *IL-34* KO mice (5, 22, 23). To confirm that this represents maximal depletion with these reagents, we conducted a study comparing 100 and 200 mg/kg of each antibody dosed individually. The same degree of depletion was seen with 100 or 200 mg/kg anti-CSF1 in the fimbria, and with 100 or 200 mg/kg of anti-IL-34 in the cortex (Figure S4). This suggests that dosing with anti-CSF1 and anti-IL-34 can lead to complete functional inhibition of their respective ligands in the CNS.

To determine whether CSF1R inhibition had an effect on the remaining microglia, we measured microglia size. Using a custom Matlab routine, a cell mask that included the soma and processes was generated for each microglia and used to calculate the size (μm^3^) of each cell. We found that in the depletion conditions that lead to the greatest loss of microglia, there was a concomitant increase in the size of the remaining microglia in both fimbria and cortex (Figure S5). This trend was clearest in the cortex, with a dose-dependent increase in the size of remaining microglia seen in animals treated with 30, 60, and 100 mg/kg of anti-IL-34. The largest increase in average size was seen in combo and PLX3397 dosed mice, the dosing conditions that lead to the greatest degree of cortical depletion (Figure S5). There was a smaller, but still significant change in microglia size seen in the fimbria in anti-CSF1, combo, and PLX3397 dosing groups (Figure S5). The increase in microglia size in brain regions showing significant microglia depletion may be due to a loss of contact with other microglia in a normal tiled array that would typically limit the territory of a given microglia's processes (34).

### Microglia Depletion Does Not Impact the Density of Other Cells Types in the Brain Parenchyma

**Figure 3:**
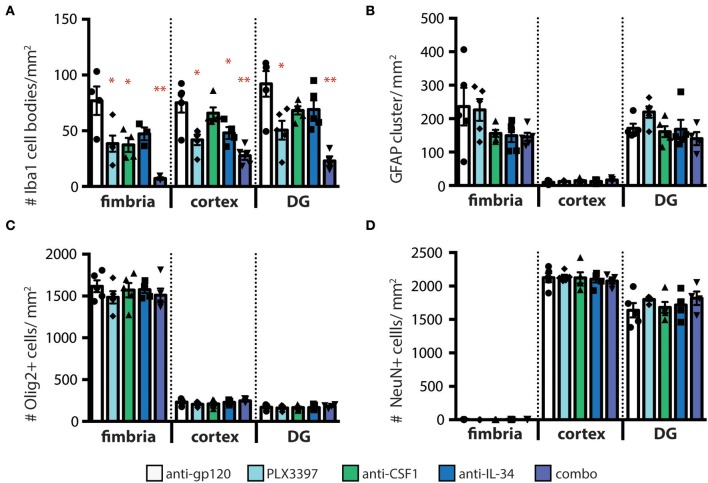
Other cell types in the brain parenchyma are not affected by regional microglia depletion with anti-IL-34 and anti-CSF1. **(A–D)** Quantification of immunohistochemistry (IHC) for cell type specific markers on brain sections from adult *CX*_3_*CR1*^*GFPki*/+^ mice dosed with anti-gp120, anti-IL-34, anti-CSF1, combo (anti-IL-34 plus anti-CSF1) dosed IP 2X per week for 3 weeks at 100 mg/kg each or PLX3397 (formulated in chow at 290 mg/kg for 3 weeks), in fimbria, cortex and dentate gyrus (DG). **(A)** Quantification of microglia density (Iba1^+^ cells). **(B)** Quantification of astrocyte density (GFAP^+^ cells). **(C)** Quantification of oligodendrocyte density (Olig2^+^ cells). **(D)** Quantification of neurons (NeuN^+^ cells). *n* = 3–6 mice per group. Data are represented as mean ± SEM, ^*^p < 0.05, ^**^p < 0.001. Data were analyzed with a one-way ANOVA with a *post-hoc* Dunnett's test.

To confirm that loss of GFP+ microglia in *CX*_3_*CR1*^*GFPki*/+^ animals corresponds to loss of microglia, we stained coronal brain sections from treated mice for the microglia marker Iba1. Similar to our findings assessing GFP+ cell density, the number of Iba1+ microglia was reduced, in a region-specific manner, in animals dosed with anti-CSF1 and anti-IL-34 antibodies (Figure 3A). Importantly, while the absolute density of microglia GFP+ and Iba+ microglia differ due to the two techniques used to measure them (see details in methods sections) the relative differences between treatment conditions are preserved. To investigate the effect of microglia depletion on other cell types in the parenchyma, we stained sections for GFAP, Olig2, and NeuN to assess densities of astrocytes, oligodendrocytes, and neurons respectively. None of the microglia depletion conditions tested caused a change in density of other cell types in the fimbria, cortex, or dentate gyrus (Figures 3B–D). Therefore, microglia depletion by CSF1 or IL-34 neutralizing antibodies does not cause a change in density of other cell types in the brain parenchyma to fill the niche left by depleted microglia, similar to the results seen by other investigators when depleting microglia in adult mice with other techniques (29). To determine whether astrocytes were becoming activated by microglia depletion, we also assessed GFAP staining intensity, as GFAP expression increases in response to insults in the brain (35). We found that GFAP expression per cell did not increase in response to microglia depletion (Figure S6).

### IL-34 and CSF1 Exhibit Differential Regional Expression that Corresponds With Regions of Ligand-Specific Microglia Depletion

**Figure 4:**
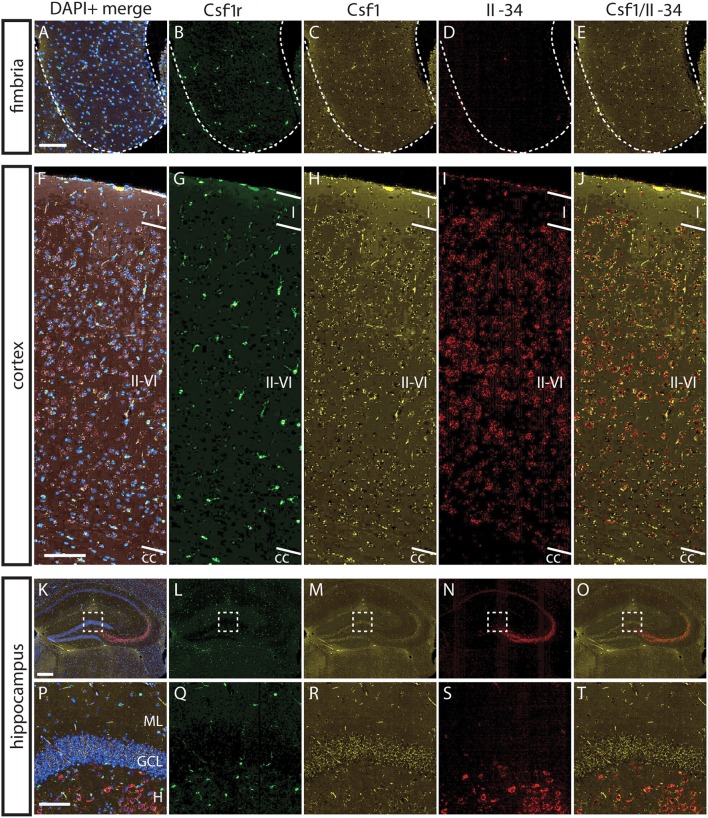
Differential expression of IL-34 and CSF1 in gray and white matter brain regions. **(A–T)** Representative images of multi-color fluorescent *in situ* hybridization (FISH) on coronal brain slices from BL6 wild type (WT) adult mice for Csf1r, Csf1, and Il-34 in the fimbria **(A–E)**, cortex **(F–J)**, and hippocampus **(K–O)**. **(P–T)** Magnification of DG from K-O (dashed box). I—cortical layer I, II–VI—cortical layers II–VI; cc, corpus callosum; DG, dentate gyrus; ML, molecular layer; GCL, granule cell layer; H, hilus. Scale bars, 100 μm for fimbria, cortex, and DG inset; 300 microns for hippocampus **(K–O)**.

To understand the basis for differential regional microglia depletion by anti-CSF1 and anti-IL-34, we ran a multi-color fluorescent *in situ* hybridization (FISH) assay capable of single molecule detection to examine the transcriptional expression pattern of each ligand and the receptor (36, 37). In adult mice, Csf1r is expressed in cells throughout the brain in a pattern consistent with microglia (Figures 4B,G,L). Both Csf1 and Il-34 are expressed in both gray and white matter regions, but at different levels relative to one another in each tissue compartment. In the fimbria, more Csf1 is expressed than Il-34 (Figures 4A–E; Csf1: 2.08% tissue area stained, Il-34: 0.03% tissue area stained). In the cortex, more Il-34 is expressed than Csf1 (Figures 4F–J; Csf1: 4.69% tissue area stained, Il-34: 9.56% tissue area stained). Both Csf1 and Il-34 are expressed throughout the hippocampus (Figures 4K–O; Csf1: 5.96% tissue area stained, Il-34: 1.66% tissue area stained), but each ligand has a distinct expression pattern within subregions of the tissue compartment. In the molecular layer Csf1 expression is higher, in the hilus Il-34 expression is higher, and in the granule cell layer only Csf1 is expressed (Figures 4P–T). It is interesting to note that the fimbria, which has the lowest overall expression levels of CSF1R ligands, is the only brain region where 10 mg/kg anti-CSF1 dosing was sufficient to deplete microglia (Figure 1), suggesting that fully blocking CSF1 binding in this brain region would require less antibody. Taken together, these data show that in gray and white matter regions, the more highly expressed ligand corresponds to the ligand-blocking antibody that can effectively deplete microglia as a single agent. In the mixed brain regions, like the hippocampus, the subregion-specific expression pattern requires both function-blocking antibodies to be dosed to significantly deplete microglia.

### CSF1, but Not IL-34, Is Required for Prenatal Microglia Development and Colonization of the Brain

**Figure 5:**
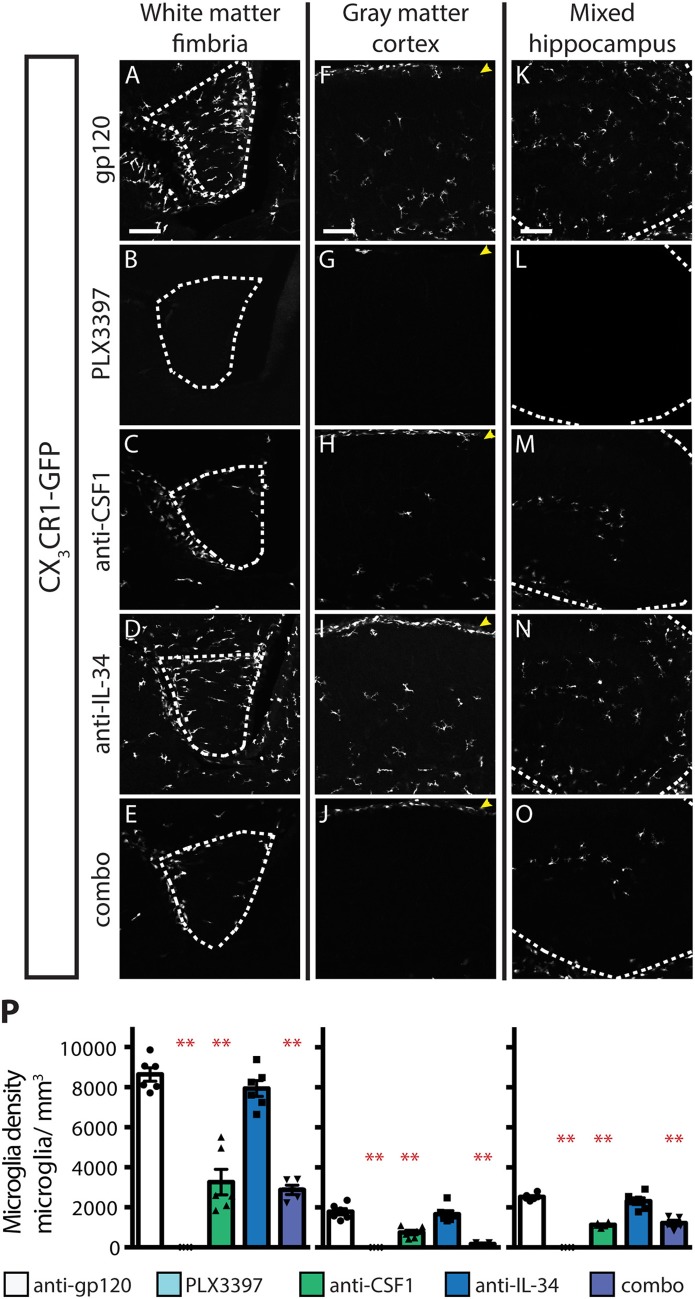
CSF1, but not IL-34, is required for microglia survival during embryogenesis. **(A–O)** Representative images of microglia in brains from P0.5 *CX*_3_*CR1*^*GFPki*/+^ pups from timed-pregnant dams treated with anti-gp120 (control IgG), anti-IL-34, anti-CSF1, combo (anti-IL-34 plus anti-CSF1) dosed IP, 100 mg/ kg each, at E6.5 and E7.5. PLX3397 dosed at 290 mg/kg of chow in timed-pregnant dams starting at E6.5 through birth. Images of fimbria **(A–E**; white matter; outlined by dashed white lines), cortex **(F–J**; gray matter; yellow arrowheads: meningeal macrophages), and hippocampus (**K–O**; outlined by dashed white line). **(P)** Quantification of microglia density in fimbria, cortex, and hippocampus. *n* = 5–6 animals/group. Data are represented as mean ± SEM, ^**^ indicates *p* < 0.0001. Data were analyzed with a one-way ANOVA with a *post-hoc* Dunnett's test. Scale bars, 100 μm.

While a requirement for CSF1R has been established for microglia development during embryogenesis (5), it is unclear whether either or both known ligands are required for microglia development. At E8-E9, microglia precursors migrate from the yolk sac and infiltrate the developing the brain (5, 38). Previous work has shown that injection of a blocking antibody to CSF1R at this developmental stage depletes yolk sac macrophages, which are precursors to CNS microglia, and leads to a near complete loss of microglia in the developing mouse brain (9). To understand the relative contributions of CSF1 and IL-34 to microglia colonization of the developing brain, timed-pregnant (TP) *CX*_3_*CR1*^*GFPki*/+^ animals were dosed with anti-gp120, or anti-CSF1 and/or anti-IL-34 at E6.5 and E7.5, and microglia density was assessed at P0.5. An additional set of TP *CX*_3_*CR1*^*GFPki*/+^ animals were dosed with PLX3397 chow from E6.5 through birth. PLX3397 dosing from E6.5 to P0.5 almost fully prevented microglia colonization of the brain (Figure 5), with a few scattered microglia present throughout the brain and a slightly greater concentration of microglia in the hypothalamus near the third ventricle. Unlike what was seen in adult mouse brain, anti-CSF1 dosing caused a significant decrease in microglia density in all brain regions assessed, including fimbria, cortex, and hippocampus, with a similar degree of depletion seen in all brain regions analyzed (Figures 5C,H,M,P; 56–63% depletion). Anti-IL-34 on the other hand, had no effect on microglia density in any region of the brain when singly dosed (Figures 5D,I,N,P). This was not simply due to clearance of the antibody, as pups born to TPs dosed with anti-IL-34 at E6.5 and E7.5 plus twice per week through the remainder of gestation still showed no microglia depletion in any brain region examined (data not shown). Combo dosing had no increased effect over dosing anti-CSF1 alone in the fimbria or hippocampus, but did significantly increase depletion compared to anti-CSF1 in the cortex (Figures 5E,J,O,P; 23% increase in depletion), revealing a small role for IL-34 in microglia survival that can presumably be compensated for by CSF1 in prenatal development.

**Figure 6:**
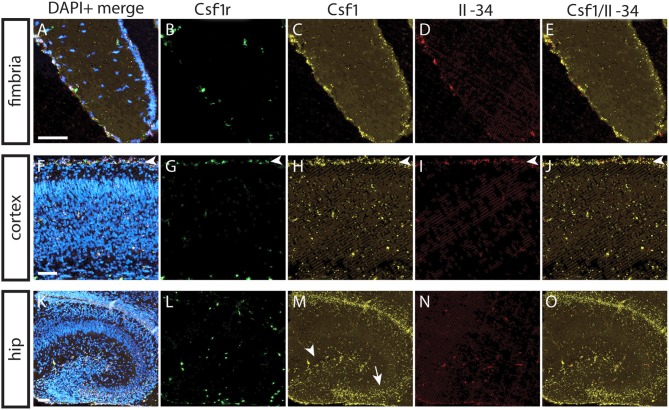
CSF1 is predominant CSF1R ligand expressed in all brain regions at P1. **(A–O)** Representative images of multi-color fluorescent *in situ* hybridization (FISH) on coronal brain slices from BL6 WT P1 mice for Csf1r, Csf1, and Il-34 in the fimbria **(A–E)**, cortex (**F–J**; arrowheads—meninges), and hippocampus **(K–O)**. **(M)** Dentate gyrus: arrowhead, CA3: arrow. Hip, hippocampus. Scale bars, 50 μm.

This CSF1 dependence at P0.5 suggested that the early postnatal expression patterns of CSF1R and its ligands might differ from those of adult mice. Using multicolor FISH we assessed the transcriptional expression patterns of CSF1R, CSF1, and IL-34 in P1 brain. In both white and gray matter regions Csf1 expression is higher than Il-34 expression (Figures 6A–J; fimbria—Csf1: 1.39% tissue area stained, Il-34: 0.11% tissue area stained; cortex—Csf1: 6.21% tissue area stained, IL-34: 0.05% tissue area stained). Both Csf1 and Il-34 are highly expressed in the meninges (Figures 6H–I; arrowheads). And in the hippocampus, expression of Csf1 is again higher than Il-34, especially in the developing DG and CA3 regions (Figures 6K–O; Csf1: 6.14% tissue area stained, Il-34: 0.18% tissue area stained). These results show that at P1, as in adult brain, the pattern of ligand expression corresponds to the pattern of anti-CSF1 and anti-IL-34 mediated microglia depletion.

### Both IL-34 and CSF1 Promote Microglial Survival in the Early Postnatal Brain, but Region-Specific Ligand Dependence Emerges After P4

**Figure 7:**
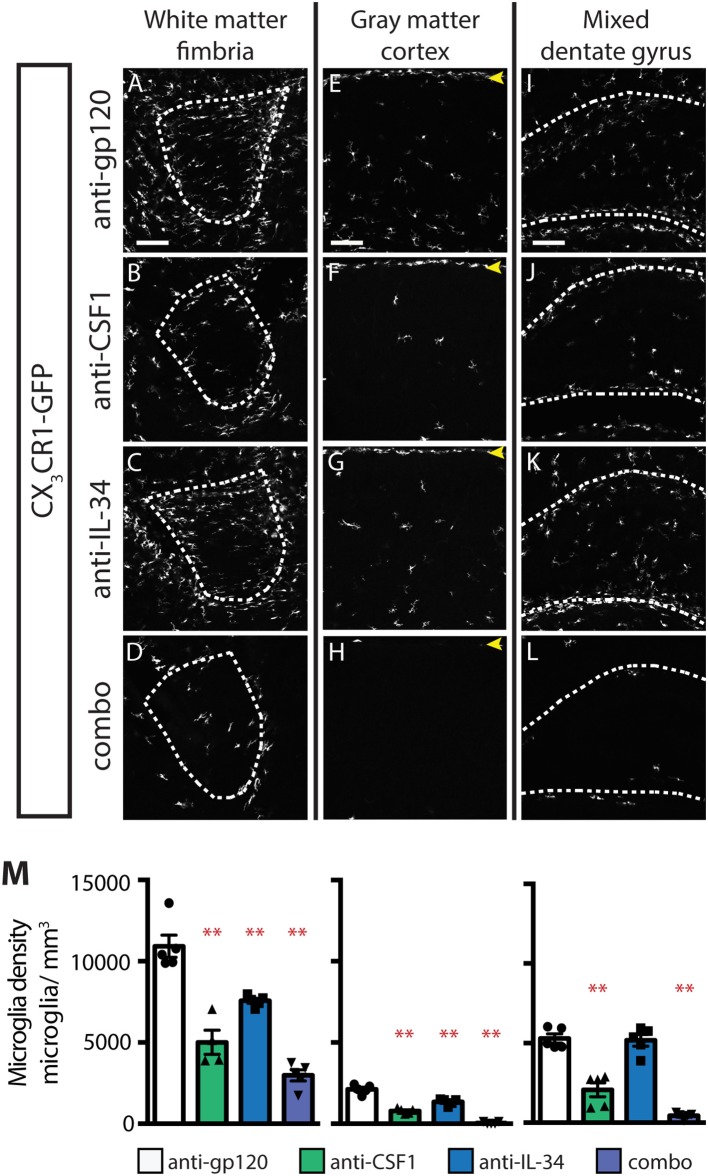
Both IL-34 and CSF1 are required for microglial survival by P4. **(A–L)** Representative images of microglia in brains from P4 *CX*_3_*CR1*^*GFPki*/+^ pups treated with anti-gp120 (control IgG), anti-IL-34, anti-CSF1, combo (anti-IL-34 plus anti-CSF1) dosed subcutaneously, 100 mg/kg each, at P0.5. Images of fimbria **(A–D**; white matter, outlined by dashed white lines), cortex (**E–H**; gray matter; yellow arrowheads: meningeal macrophages), and dentate gyrus (**I–L**; outlined by dashed white lines). **(M)** Quantification of microglia density in fimbria, cortex, and dentate gyrus. *n* = 4–5 animals/group. Data are represented as mean ± SEM, ^**^ indicates *p* < 0.0001. Data were analyzed with a one-way ANOVA with a *post-hoc* Dunnett's test. Scale bars, 100 μm.

While IL-34 is not required for microglia survival at P0.5, it is clearly required, particularly in gray matter, in adult brain (Figure 1). To understand when during postnatal development IL-34 is first required for microglia survival, we dosed P0.5 *CX*_3_*CR1*^*GFPki*/+^ pups, subcutaneously, with anti-gp120, anti-CSF1, anti-IL-34, or combo, and assessed microglia density in the brain at P4. In the fimbria and cortex, brains from anti-IL-34 dosed pups showed significant microglia depletion (Figures 7A–H,M; anti-IL-34: ~31% depletion). Anti-CSF1 dosing caused significantly increased depletion compared to anti-IL-34 dosed singly in fimbria and cortex, and combo dosing leads to a significant increase in depletion over either antibody dosed individually (Figures 7A–H,M, anti-CSF1: ~54% depletion, combo: ~73% depletion). At P4, anti-IL-34 did not significantly deplete microglia in the DG but anti-CSF1 did, as was seen in DG at P0.5 (Figures 7I–L,M). Unlike P0.5 DG, combo dosed mice showed a significant increase in microglia depletion over anti-CSF1 dosed singly in P4 brain (Figures 7L,M). Interestingly, at P4, meningeal macrophages are not depleted with either anti-IL-34 or anti-CSF1 dosed singly, but they were significantly depleted with combo dosing (Figures 7E–H, arrows). This suggests a previously unknown role for IL-34, which had been reported to be required only for microglia and Langerhan's cell survival (22, 24). Together, these data suggest that IL-34 can promote microglia survival in both gray and white matter brain regions by P4, and that the gray vs. white matter specificity of IL-34 and CSF1 observed in adult animals is likely established at a later point in development.

## Discussion

In our current study we have made several important advances in understanding the roles of IL-34 and CSF1 in microglia development and maintenance. We showed that microglia can be differentially depleted in gray and white matter by peripheral dosing of anti-IL-34 and anti-CSF1, respectively; and we showed that this differential in microglia depletion is explained by the different expression patterns of IL-34 and CSF1 in gray and white matter regions of the brain. Additionally, we showed that there is a transition from CSF1 being the sole CSF1R ligand required for microglial maintenance during embryonic development, to a role for both CSF1 and IL-34 in microglia maintenance and proliferation during early postnatal development. Finally, we showed that the regional differences in expression of IL-34 and CSF1 in gray and white matter develop postnatally, after P1.

Reports of the microglial densities in CSF1 null and IL-34 null mice have been either incomplete or somewhat contradictory due to a variety of potential factors, including differences in housing that could affect microbiota, and alter microglia (39), differences in strain background, as well as technical differences in how experiments were performed. IL-34^lacZ/lacZ^ mice are reported to have wild-type (WT) microglia density at birth (22), or a nearly complete loss of microglia at P2 (24), and varying degrees of microglia depletion in gray and white matter regions throughout the brain in adult mice. Our embryonic anti-IL-34 dosing experiments confirm that IL-34 signaling is dispensable for microglia survival in the brain prior to P1 (Figure 5) (22). In adult mice, the varied reports of microglia depletion in IL-34^lacZ/lacZ^ mice, especially the microglial loss seen in white matter regions that is not replicated with anti-IL-34 treatment, may be due to a developmental effect of IL-34 loss. As IL-34 supports microglia maintenance in the white matter at P4, it is possible that in the IL-34^lacZ/lacZ^ mice, microglia fail to localize to white matter tracts in sufficient numbers during postnatal development, and/or are not stimulated to proliferate properly to achieve normal adult microglial densities. In the CSF1^op/op^ mouse, there are no published reports of microglia density prior to 3 weeks old, possibly due to the common use of failure of teeth to erupt at 3 weeks to identify CSF1^op/op^ from CSF1^WT/op^ littermates (25). At 3–4 weeks old CSF1^op/op^ mice are reported to have reduced microglia density (5, 40) that may (25) or may not (5, 23) be recovered as mice age. Our results confirm what has been suggested, but not previously shown, that CSF1 is required for microglia survival during embryonic development (Figure 5) (5). No decrease in microglia density is seen in the cortex in adult mice dosed with anti-CSF1, suggesting that the reported CSF1^op/op^ reduced microglia density phenotype may be driven by a developmental defect in microglia migration or proliferation.

Our results concerning the expression pattern of CSF1, IL-34, and CSF1R also diverge from previous reports. Studies of the expression pattern of CSF1R and its ligands have suggested that CSF1 and IL-34 have complementary expression patterns in the mouse cortex, with CSF1 expression restricted to layer VI in P2 and P20 mice, and IL-34 expressed in layer V at P2 and layers I-V at P20 (40, 41). This differs from our finding that both ligands are expressed throughout the cortical layers in P1 and in adult mice, but with CSF1 expressed much more strongly than IL-34 at P1, and IL-34 expressed to a greater degree throughout adult cortex than CSF1 (Figures 4, 6). These data are supported by our microglia depletion data in both embryonic and adult mice (Figures 1, 5), and is consistent with a recent study describing functional consequences of regional expression of CSF1R ligands (42). These difference in reported expression patterns may be due to the difference in sensitivity of the detection methods, for example the ISH method used in this study is capable of single molecule resolution of targets (36).

Recently, it has become clear that different subpopulations of microglia exist, with distinct expression profiles being identified for microglia in various regions of the brain, and at different points in development (43–45). Our data support the idea that gray and white matter microglia may be distinct populations, with CSF1 playing a greater role in supporting white matter microglia maintenance, and IL-34 playing a greater role in supporting gray matter microglia maintenance (Figure 1). While both ligands can support microglia survival, they have distinct but overlapping binding sites on CSF1R, and binding of each ligand has been shown to cause different degrees of receptor activation and differential downstream phosphorylation of targets including MAPK and Src family members (19, 46). Expression profile analysis of purified white and gray matter microglia should provide an intriguing window into the downstream effects of this differential phosphorylation and may soon lead to molecular signatures being identified for different microglia subtypes.

It has been well-established that CSF1R signaling is required for survival of adult microglia. *Csf1r*^−/−^ mice have almost no microglia, and administration of small molecule inhibitors to CSF1R leads to the loss of almost all brain microglia (5, 26, 29, 30). CSF1 and IL-34 are currently thought to be the only ligands for CSF1R, and their phenotypes of partial microglia loss had been proposed to be additive to the receptor loss phenotype (40). If this were correct, we would predict that combo dosing of anti-CSF1 and anti-IL-34 would deplete microglia to a similar degree as is seen with the CSF1R SMI PLX3397 in all brain regions. Instead, we see that while combo dosing depletes to a similar degree as PLX3397 in white matter regions, combo dosing fails to deplete microglia to the same degree as the SMI in gray matter (Figure 1 and Figure S1). PLX3397 is small molecule that inhibits receptor tyrosine kinases other than CSF1R (e.g., Flt3 and cKit) (47), which may contribute to the discrepancy between PLX3397 and anti-CSF1/anti-IL-34 combo treated animals. However, this raises the question of whether an additional, as of yet unidentified ligand for CSF1R exists that is expressed in the gray matter of adult brain. Receptor-ligand screening could shed light on this question.

Depleting sub-populations of microglia using function-blocking antibodies to CSF1 and IL-34 has the potential to provide novel insights into the roles of microglia in both development and disease. During development, microglia are understood to play important roles in varied processes, from an embryonic role in establishing correct brain architecture to postnatal roles in pruning excess synapses (9, 12, 13). Blocking CSF1 or IL-34 in studies of synapse refinement postnatally is a novel way to dissect the role of microglia in intact animals, after brain architecture has been established, allowing an assessment of the specific roles of sub-populations of microglia in these processes (Figure 5). In disease, a role for microglia has been suggested in disparate conditions from neurodegenerative disorders, such as Alzheimer's disease and amyotrophic lateral sclerosis (ALS), to schizophrenia and depression (48–52). In mouse models of these diseases, depleting white or gray matter microglia may allow greater insight into whether these cells are playing an active role in driving pathology and may indicate whether depletion of specific microglia populations can be an effective disease-modifying therapy.

## Methods

### Animal Models

All animal procedures were carried out with the Institutional Animal Care and Use Committee's (IACUC) approval in accordance with the institution's ethical guidelines. All animals were derived within the C57Bl6 murine background. *CX*_3_*CR1*^*GFPki*/+^ mice, a line expressing GFP in all microglia, were generated and characterized previously (32). For timed-pregnant (TP) dams, the day of vaginal plug was considered E0.5.

### Microglia Depletion

Anti-IL-34 and anti-CSF1 mouse IgG2a monoclonal antibodies were generated in-house and described elsewhere (31). Anti-gp120 IgG control (Genentech), anti-IL-34, and anti-CSF1 were dosed at 100 mg/kg and combo (anti-IL-34 plus anti-CSF1) were dosed at 100 mg/kg of each antibody unless otherwise stated in the text. For adult analysis, *CX*_3_*CR1*^*GFPki*/+^ animals between 8 and 12 weeks old were dosed with antibody by intraperitoneal injection (IP) twice per week for 3 weeks unless otherwise stated in the text. For P0.5 analysis, TPs were dosed IP with antibody at E6.5 and E7.5. For P4 analysis, P0.5 pups were dosed subcutaneously with antibody into the dorsal skin fold. PLX3397 (Pexidartinib) was purchased from ChemShuttle (Hayward, CA) and formulated in AIN-93G chow by Envigo (Hayward, CA) at 290 mg/kg of chow. PLX3397 chow was dosed *ad libitum* for 3 weeks for adult analysis, and from E6.5 to P0.5 for P0.5 analysis.

### Analysis of Microglia in the Brain

Adult animals were anesthetized and perfused with 10 mL of PBS followed by 10 mL of 4% PFA + 10% sucrose in PBS. Brains were collected and fixed in 4% PFA + 10% sucrose in PBS overnight. Post fixation, brains were embedded in 1.5% agarose in 35 mm dishes that were filled with PBS for *en bloc* imaging of the cortex. To image other brain regions 300 μm coronal sections were cut on a vibratome. P0.5 pups were anesthetized on ice then decapitated. Brains were collected and fixed in 4% PFA + 10% sucrose in PBS overnight. P4 pups were anesthetized on ice then perfused with 5 mL of 4% PFA + 10% sucrose in PBS. Brains were collected and fixed in 4% PFA + 10% sucrose in PBS overnight. Both P0.5 and P4 brains were embedded in 5% agarose and 150 or 300 micron coronal sections were cut on a vibratome. GFP+ microglia were imaged using a 2-photon microscope (Prairie Technologies Ultima IV microscope powered by a Spectra Physics MaiTai DeepSee laser). Microglia were visualized under a 20 × NA 1.0 objective (Olympus), with a field of view 1,024 × 1,024 pixels at 0.592 μm/pixel, and z-step size of 1.5 μm, using 910 nm laser wavelength and 512/630-25 nm dual-band bandpass filter (Semrock) placed before the GaAsP detector. Individual microglia were identified in these image stacks using a custom image analysis routine in Matlab (Mathworks). Microglia density was calculated per animal as the total number of microglia divided by the image volume. For size analysis, individual microglia were identified and size was calculated using a custom image analysis routine in Matlab (Mathworks).

### Analysis of Microglia Density in the Spinal Cord

After PFA perfusion, spinal cords were removed via laminectomy. Spinal cords were embedded in 5% agarose and 300 micron cross sections were cut on a vibratome. Imaging was done as described for brain microglia, but with a 10 × NA 0.6 objective (Olympus), with a field of view 1,024 × 1,024 pixels at 2.3649 μm/pixel. Individual microglia were identified using a custom image analysis routine in Matlab (Mathworks). Number of microglia per area was calculated per animal as the total number of microglia divided by the image area.

### LC-MS Assay for the Determination of PLX3397 Concentrations in Brain

The concentrations of PLX3397 in plasma and brain were determined by a non-validated liquid chromatography–tandem mass spectrometry (LC-MS/MS) assay using indomethacin as the internal standard (IS). Brain tissue was homogenized with 5 fold of water. A 25 uL aliquot of brain homogenate sample was pipetted into a 96-well plate containing 5 uL of DMSO. The samples were then crashed using 200 μL of acetonitrile containing 150 ng/mL of indomethacin. After vortexing at 1,000 rpm for 1 min, the samples were centrifuged at 13,000 rpm for 10 min at room temperature (RT). Next, 100 μL of supernatant was transferred to a clean 96 deep-well plate and diluted further with 150 μL of water. Finally, 1 μL of this final mixture was injected onto the analytical column Kinetex XB-C18 column (50 × 2.1 mm, 2.6 μm, Phenomenex, Torrance, CA).

Sample analysis was carried out with a Shimadzu Nexera (Columbia, MD) coupled to an API 5500 Q trap Mass Spectrometer (AB Sciex, Foster City, CA) equipped with a turbo-electrospray interface in positive ionization mode. The aqueous mobile phase was water with 0.1% formic acid (A) and the organic mobile phase was acetonitrile with 0.1% formic acid (B). The gradient was as follows: 10% B for the first 0.1 min, increased to 90% B from 0.1 to 0.6 min, maintained at 90% B from 0.6 to 0.8 min, decreased to 10% B at 0.81 min, and maintained at 10% B from 0.81 to 1.00 min. The flow rate is 1.2 ml/min and the cycle time (injection to injection) was approximately 1.2 min. Quantitation was carried out using the multiple reaction monitoring (MRM) transition *m/z* 418.035 257.000 for PLX3397 and *m/z* 358 → 139 for the IS. The lower limits of quantitation of the assay were 0.0073 μM for PLX3397. The optimized instrument conditions were as follows: source temperature, 550°C; curtain gas, 35 psi; nebulizing (GS1), 55 psi; heating (GS2), 60 psi; collision energy (CE), 51 V for PLX3397 and 25 V for the IS. LC-MS/MS data were acquired and processed using Analyst software (v1.6.2 Applied Biosystems/MDS Sciex, Canada). The quantitation of the assay employed a calibration curve, which was constructed by plotting the analyte/internal standard peak area ratios vs. the nominal concentration of each analyte with a weighted 1/*x* quadratic regression.

### PK Analysis

The concentration-time data from individual animals were tabulated using WinNonlin^®^ version 6.4 (Pharsight; Mountain view, CA, USA).

### Histological Analysis

Immunohistochemistry was performed on 5 μm FFPE (Formalin-Fixed Paraffin Embedded) mouse brain sections for GFAP (Cat# Z0334, Rabbit Polyclonal, Dako, Carpinteria, CA), NeuN (Rabbit Polyclonal, Cat# ab104225, Abcam, Cambridge, MA), Olig2 (Rabbit Polyclonal, Cat#AB9610, Millipore, Billerica, MA) and Iba-1 (Rabbit Polyclonal, Cat# 019-19741, Wako Chemicals, Richmond, VA). GFAP immunostaining was performed on a Dako autostainer. For GFAP, brain sections were pre-treated with Proteinase K (Cat# S3020, Dako) for 5 min at RT. Sections were rinsed in TBST (Tris-Buffered Saline and Tween-20) for 5 min. Sections were quenched for endogenous peroxidase in 3% hydrogen peroxide solution for 4 min at RT before blocking in 10% Normal Goat serum in 1X PBS for 30 min at RT. Tissue sections were incubated with 2.07 μg/ml of GFAP antibody diluted in blocking buffer for 1 h at RT followed by detection with Powervision Poly-HRP anti-rabbit (Cat# PV6119, Leica, Buffalo Grove, IL) and DAB (Thermo Scientific, Waltham, MA). Naïve rabbit polyclonal antibody (Cat#2729S, Cell Signaling Technologies, Danvers, MA) was used as isotype control. Further, sections were incubated for 30 min at RT in biotinylated rabbit anti-rat secondary antibody (Vector Labs, Burlingame, CA) diluted to 2.5 μg/ml in blocking buffer and DAB-based signal was detected with ABC-HRP (Vector Labs). Iba-1, NeuN and Olig2 IHC staining were performed on Discovery XT autostainer (Ventana, Tucson, AZ). For Iba-1, the FFPE tissue sections were pretreated with Ventana CC1 mild antigen retrieval for Iba-1 (0.25 μg/ml) whereas tissue sections were pretreated with CC1 standard antigen retrieval for NeuN (1:3,000 dilution) and Olig2 (1 μg/ml). Naïve rabbit polyclonal was used as isotype control. Detection was done using OmniMAP HRP anti-rabbit polymer (Cat# 760-4311) and Ventana ChromoMap DAB detection kit (Cat# 760-159). Tissues were counterstained with Ventana Hematoxylin II. Digital whole slide images were acquired using the Hamamatsu Nanozoomer 2.0-HT automated slide scanning system at 200× magnification. Image analysis was performed using Mathworks software (Matlab version R2016b). Whole tissue and immunohistochemically stained area were identified using intensity/RGB thresholding and morphological filtering. Positive cell bodies were automatically counted using color thresholds and minima-controlled watershed algorithm with morphological operations (53). Regions of interest (ROI) consisting of the cortex, dentate gyrus, and hippocampal fimbria were manually drawn. The number of cell bodies or stained area was normalized to the whole tissue section or ROI area. Cell intensity was measured in 8-bit grayscale from lightest to darkest staining in 5 unit bins.

### *In situ* Hybridization

RNA *in situ* hybridization for Csf1r, Csf1, and Il-34 mRNA was performed on the Leica Bond Rx automated staining platform using the RNAscope^®^ LS Multiplex Fluorescent Reagent Kit (Cat. 322800) according to the manufacturer's instructions (Advanced Cell Diagnostics, Inc., Newark, CA). Briefly, 5 μm FFPE tissue sections were pretreated with heat and protease prior to hybridization with the target oligo probes. Optimization was performed to identify the best pre-treatment conditions for the samples. Final conditions were 15 min BOND Epitope Retrieval Buffer 2 (ER2) at 88°C and 15 min RNAscope^®^ 2.5 LS Protease III at 40°C for all samples except the mouse multi-tissue array, which was run at 15 min ER2 at 95°C and 15 min Protease III at 40°C. Preamplifier, amplifier, and horseradish peroxidase (HRP)-labeled oligos were then hybridized in a series of sequential amplification steps, and fluorescence reagents were covalently deposited adjacent to the immobilized HRP through an enzyme catalysis reaction. Each sample was quality controlled for RNA integrity with an RNAscope^®^ probe specific to Polr2a/Ppib/Ubc mRNA and for background with a probe specific to bacterial dapB mRNA. Specific RNA staining signal was identified as punctate fluorescent dots. Samples were also counterstained with a DAPI nuclear stain. RNAscope probes and fluorophores used in this study were Csf1r (Channel 1, fluorescein, Cat. 428198), Csf1 (Channel 2, cyanine 3, Cat. 315628-C2), and Il34 (Channel 3, cyanine 5, Cat. 428208-C3). Digital whole slide images were acquired using the Hamamatsu Nanozoomer 2.0-HT automated slide scanning system at 200× magnification. ISH staining intensity was analyzed using FIJI (54).

### Statistical Analysis

All data is presented as mean ± SEM. For microglia density analysis and IHC cell density analysis statistical significance was assessed by One-way ANOVA with *post-hoc* Dunnett's test for comparison across multiple groups. For analysis of distribution of GFAP staining intensity, statistical significance was assessed by Kolmogorov-Smirnov tests between control and depletion conditions.

## Data Availability

All datasets generated for this study are included in the manuscript/Supplementary Files.

## Ethics Statement

The animal study was reviewed and approved by Genentech Institutional Animal Care and Use Committee.

## Author Contributions

CE-N and RW designed the study and experiments. CE-N, OF, AZ, and NS contributed to the data collection and analysis. AZ provided the reagents. CE-N and RW wrote the paper. All authors discussed and commented on the manuscript.

### Conflict of Interest Statement

The authors declare that the research was conducted in the absence of any commercial or financial relationships that could be construed as a potential conflict of interest.
