# Supplementary material for: CSF1R Ligands IL-34 and CSF1 Are Differentially Required for Microglia Development and Maintenance in White and Gray Matter Brain Regions
Source: Front Immunol. 2019 Sep 20;10:2199. doi: 10.3389/fimmu.2019.02199 (PMC6764286; doi:10.3389/fimmu.2019.02199)
Supplement: Supplementary file 1 [file Data_Sheet_1.pdf]

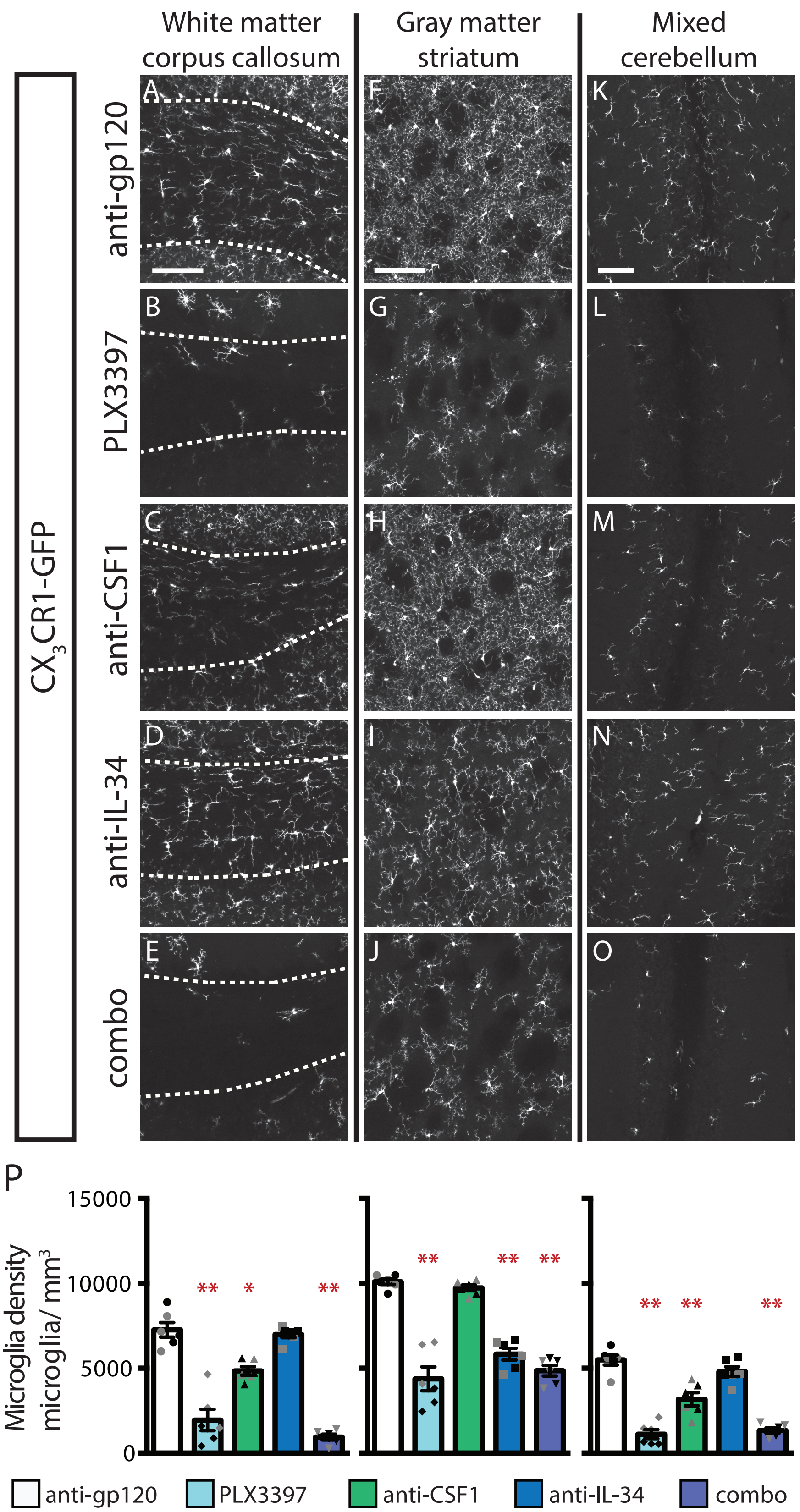

White matter corpus callosum
Gray matter striatum
Mixed cerebellum
CX_3CR1-GFP
anti-gp120
PLX3397
anti-CSF1
anti-IL-34
combo
A
B
C
D
E
F
G
H
I
J
K
L
M
N
O
P
Microglia density microglia/ mm³
15000
10000
5000
0
**
*
**
**
**
**
**
**
**
anti-gp120
PLX3397
anti-CSF1
anti-IL-34
combo

Figure S1 (related to Figure 1). The pattern of CSF1R ligand-driven microglia depletion is consistent in gray, white, and mixed brain regions.

(A-E) Representative images of microglia in brains from adult *CX3CR1*$^{GFPki/+}$ mice treated with anti-gp120 (control IgG), anti-IL-34, anti-CSF1, combo (anti-IL-34 plus anti-CSF1) dosed IP, 2X per week for 3 weeks at 100 mg/kg each or PLX3397 (formulated in chow at 290 mg/kg for 3 weeks). Images of corpus callosum (A-E; white matter; outlined by dashed white lines), striatum (F-J; gray matter), and cerebellum (K-O; cerebellar folia).

(P) Quantification of microglia density in corpus callosum, striatum, and cerebellum.

n = 6 animals/group, 3 males and 3 females. Males indicated by black symbols, females by gray symbols. Data are represented as mean ± SEM, * indicates $p < 0.05$, ** $p < 0.001$. Data were analyzed with a one-way ANOVA with a post-hoc Dunnett's test. Scale bars, 100 μm.

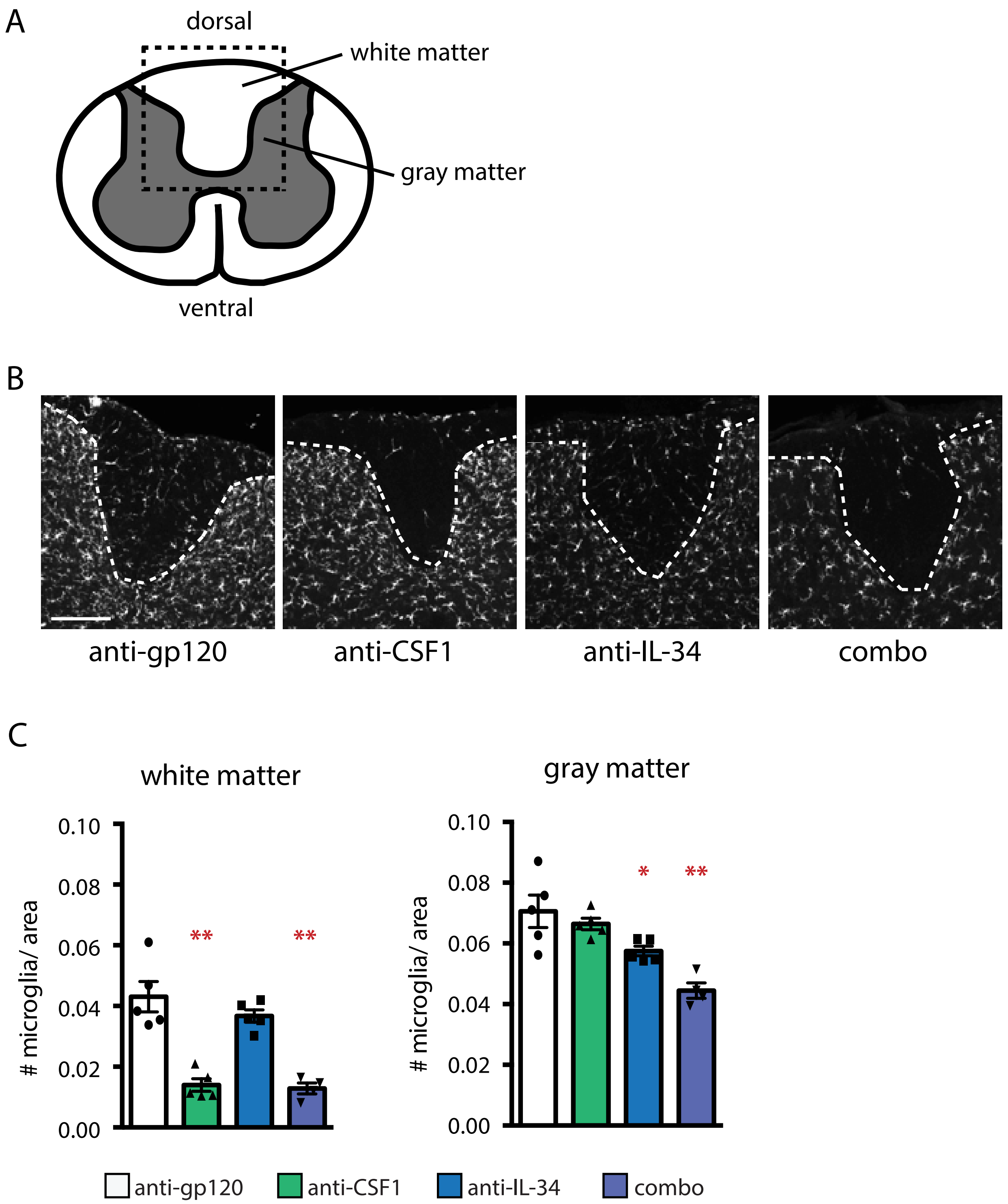

A
dorsal
white matter
gray matter
ventral
B
anti-gp120
anti-CSF1
anti-IL-34
combo
C
white matter
gray matter
microglia/ area
0.10
0.08
0.06
0.04
0.02
0.00
**
*
anti-gp120
anti-CSF1
anti-IL-34
combo

Figure S2 (related to Figure 1). Dosing with function-blocking antibodies to IL-34 and CSF1 results in differential microglial depletion in white and gray matter in the spinal cord.

(A) Diagram of cross section of the mouse lumbar spinal cord. Dashed outline indicates region imaged.

(B) Representative images of microglia in lumbar spinal cord from adult *CX3CR1*$^{GFPki/+}$ mice treated with 100 mg/kg anti-gp120 (control IgG), 100 mg/kg anti-IL-34, 60 mg/kg anti-CSF1, combo (100 mg/kg anti-IL-34 plus 60 mg/kg anti-CSF1) dosed IP, 2X per week for 3 weeks.

(C) Quantification of microglia density in the dorsal column (white matter) and dorsal horn (gray matter). Anti-CSF1 or combo dosing significantly depletes microglia in the white matter and anti-IL-34 or combo significantly depletes microglia in the gray matter. n = 5 animals/group, all males. Data are represented as mean ± SEM, * indicates $p < 0.05$, ** $p < 0.005$. Data were analyzed with a one-way ANOVA with a post-hoc Dunnett's test. Scale bar, 200 μm.

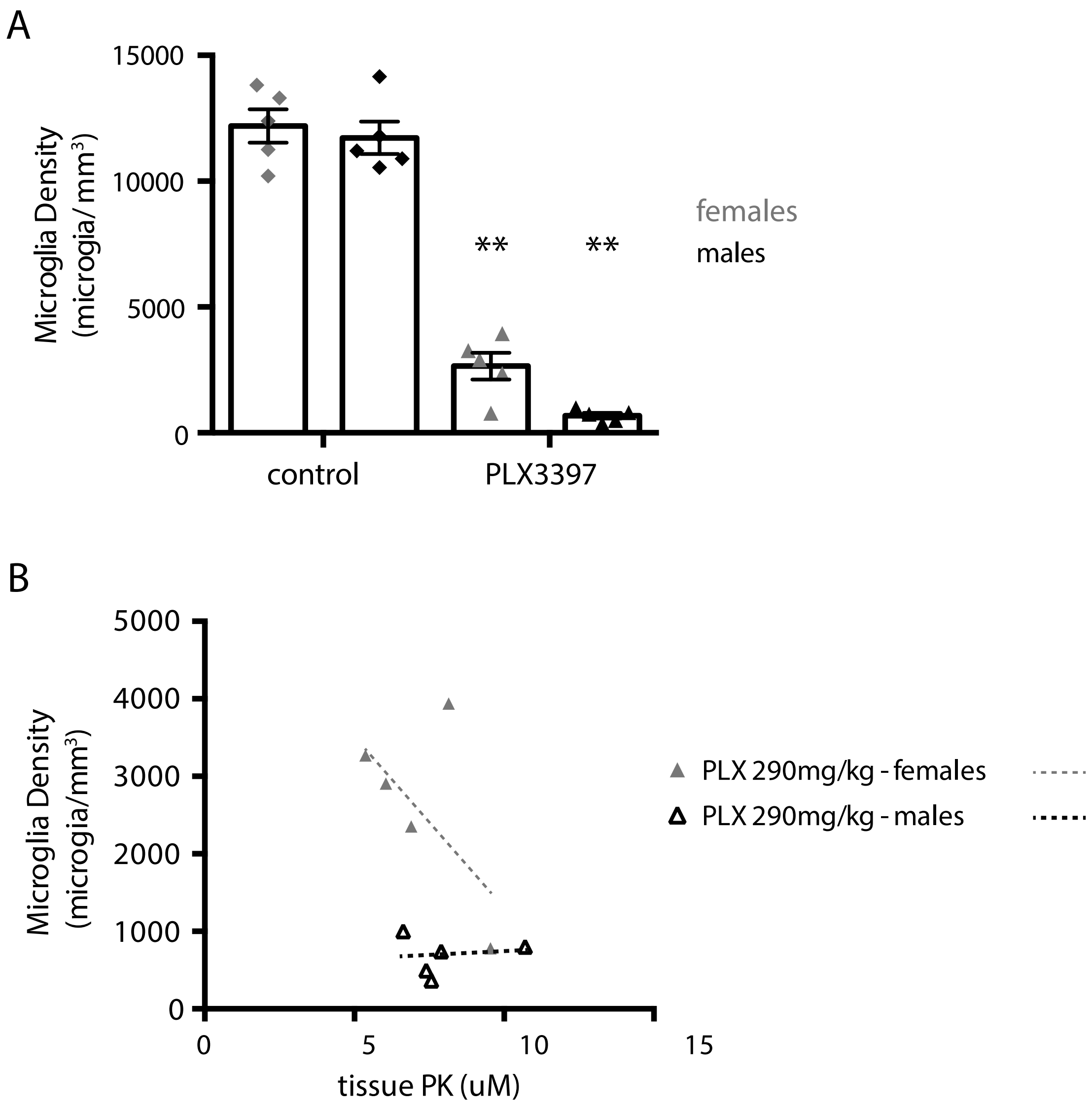

A
15000
10000
5000
0
Microglia Density (microgia/ mm3)
**
**
females
males
control
PLX3397
B
5000
4000
3000
2000
1000
0
Microglia Density (microgia/mm3)
PLX 290mg/kg - females
PLX 290mg/kg - males
0
5
10
15
tissue PK (uM)

Figure S3 (related to Figure 1). Reduced efficacy of PLX3397 in depleting microglia in female mice is caused by a sex-specific difference in drug response.

(A) Cortical microglia density in adult male and female *CX3CR1*$^{GFPki/+}$ mice dosed with control or PLX3397 chow for 3 weeks.

(B) Scatterplot of microglia density (Y-axis) and total brain tissue concentration of PLX3397 (X-axis) in male and female mice dosed with control or PLX3397 chow for 3 weeks.

n = 5 animals/ group. Data (A) are represented as mean ± SEM, ** indicates $p < 0.0001$, ** $p < 0.005$, and were analyzed with a one-way ANOVA with a post-hoc Dunnett's test.

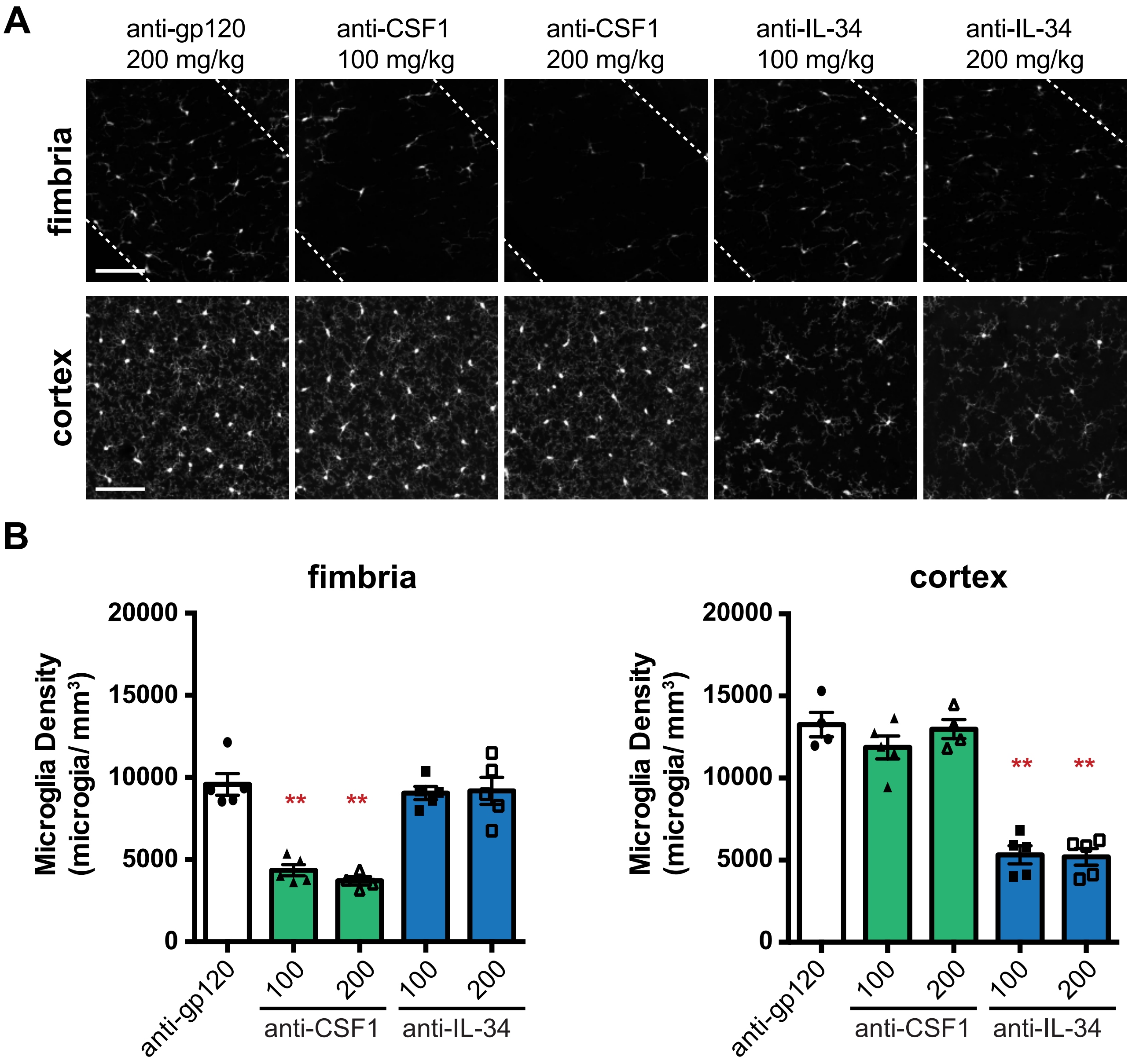
A
anti-gp120
200 mg/kg
anti-CSF1
100 mg/kg
anti-CSF1
200 mg/kg
anti-IL-34
100 mg/kg
anti-IL-34
200 mg/kg
fimbria
cortex
B
fimbria
cortex
Microglia Density
(microglia/ mm³)
20000
15000
10000
5000
0
**
anti-gp120
100
200
anti-CSF1
anti-IL-34

Figure S4 (related to Figure 2). Maximal depletion of brain microglia is achieved with 100 mg/kg dosing of anti-CSF1 and anti-IL-34.

(A) Representative images of microglia in fimbria and cortex in brains of adult $CX3CR1^{GFPki/+}$ mice treated with anti-gp120 (control IgG) at 200 mg/kg, anti-IL-34 at 100 or 200 mg/kg, or anti-CSF1 at 100 or 200 mg/kg dosed IP, 2X per week for 3 weeks.

(B) Quantification of microglia density in the fimbria and cortex.

n = 5 animals/group, mixed genders. Data are represented as mean ± SEM, ** indicates $p < 0.005$. Data were analyzed with a one-way ANOVA with a post-hoc Dunnett's test. Scale bar, 100 µm.

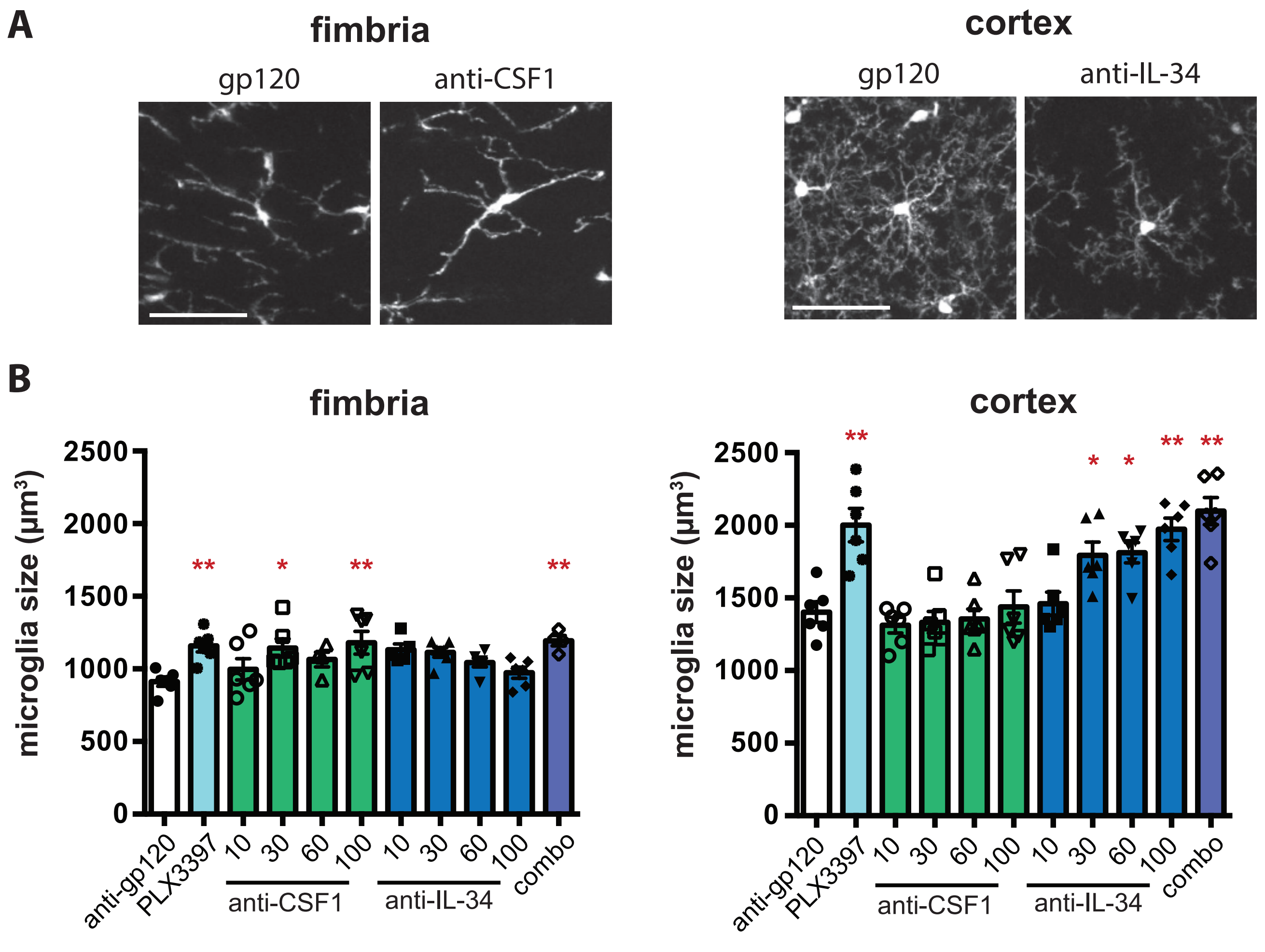
A
fimbria
gp120
anti-CSF1
cortex
gp120
anti-IL-34
B
fimbria
microglia size (µm³)
2500
2000
1500
1000
500
0
**
*
**
**
anti-gp120
PLX3397
10
30
60
100
10
30
60
100
combo
anti-CSF1
anti-IL-34
cortex
microglia size (µm³)
2500
2000
1500
1000
500
0
**
*
*
**
**
anti-gp120
PLX3397
10
30
60
100
10
30
60
100
combo
anti-CSF1
anti-IL-34

Figure S5 (related to Figure 2). Microglia size increases in response to reduced microglia density.

(A) Representative images of microglia in the fimbria and cortex of adult *CX3CR1*$^{GFPki/+}$ mice treated with 100 mg/kg anti-gp120, anti-CSF1 or anti-IL-34 dosed 2X per week for 3 weeks.

(B) Quantification of average microglia size in adult *CX3CR1*$^{GFPki/+}$ mice dosed with anti-gp120 100 mg/kg, anti-IL-34 ranging from 10-100mg/kg, anti-CSF1 ranging from 10-100mg/kg, combo (anti-CSF1 100 mg/kg + anti-IL-34 100 mg/kg), dosed IP, 2X per week for 3 weeks, or dosed with PLX3397 chow for 3 weeks.

n = 6 animals/group, 3 males and 3 females. Data are represented as mean ± SEM, * indicates $p < 0.05$, ** $p < 0.001$. Data were analyzed with a one-way ANOVA with a post-hoc Dunnett's test. Scale bars, 50 µm.

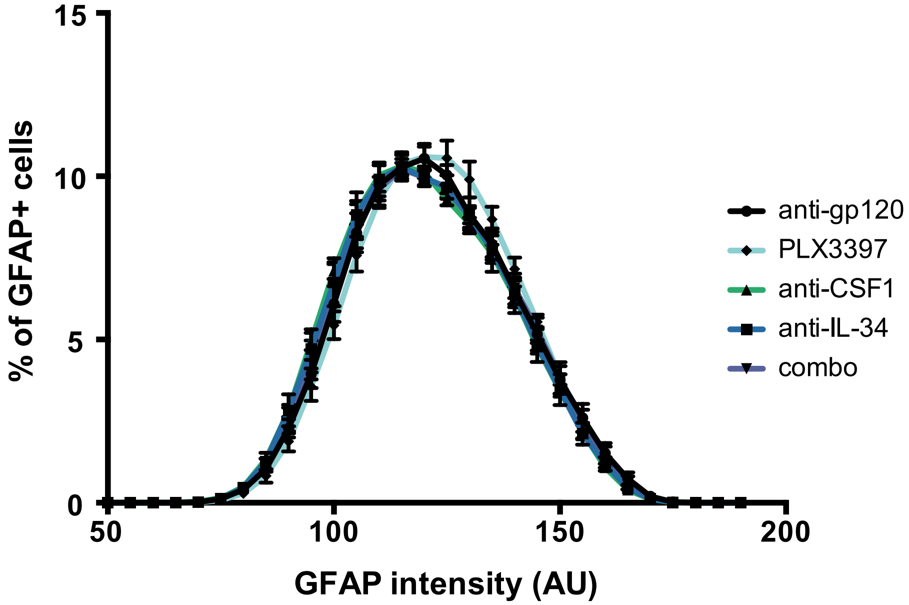
15
10
5
0
% of GFAP+ cells
50
100
150
200
GFAP intensity (AU)
anti-gp120
PLX3397
anti-CSF1
anti-IL-34
combo

Figure S6 (related to Figure 3). Microglia depletion does not cause activation of astrocytes.

Plot of percentage of GFAP positive cells (Y-axis) versus intensity of GFAP expression per cell (X-axis). n = 6 animals/group, 3 males and 3 females. Data are represented as mean ± SEM. Data were analyzed with Kolmogorov-Smirnov tests between control and depletion conditions.

Supplemental Experimental Procedures

Analysis of microglia density in the spinal cord

After PFA perfusion, spinal cords were removed via laminectomy. Spinal cords were embedded in 5% agarose and 300 micron cross sections were cut on a vibratome. Imaging was done as described for brain microglia, but with a 10x NA 0.6 objective (Olympus), with a field of view 1024 x 1024 pixels at 2.3649 μm/pixel. Individual microglia were identified using a custom image analysis routine in Matlab (Mathworks). Number of microglia per area was calculated per animal as the total number of microglia divided by the image area.
